# Editorial: Inflammation and immune factors in venous thromboembolism

**DOI:** 10.3389/fimmu.2025.1616253

**Published:** 2025-05-06

**Authors:** Steven P. Grover, Alexander Brill

**Affiliations:** ^1^ The University of North Carolina (UNC) Blood Research Center, Division of Hematology, University of North Carolina, Chapel Hill, NC, United States; ^2^ Department of Cardiovascular Sciences, College of Medicine and Health, School of Medical Sciences, University of Birmingham, Birmingham, United Kingdom

**Keywords:** VTE, deep vein thrombosis, inflammation, neutrophils, immune system

This collection of articles provides a comprehensive and timely overview of the multifaceted roles that immune and inflammatory mechanisms play in thrombus development and progression, highlights several critical aspects of the role of inflammation in venous thrombosis, and contributes significantly to the evolving understanding of venous thromboembolism (VTE) as not only a coagulation disorder but a complex immuno-inflammatory condition. Taken together, the studies presented in this Research Topic offer important mechanistic insights and set the stage for future investigations that bridge vascular biology, immunology, and thrombosis research.

A central theme linking inflammation and immunity to VTE is the concept of immunothrombosis - the idea that the immune and coagulation systems are intricately linked in a host-defense strategy that also has pathological consequences. This paradigm, originally proposed in the context of infection (1), is increasingly being applied to sterile inflammatory conditions such as postoperative thrombosis, cancer-associated thrombosis, and thrombosis in autoimmune diseases. In these settings, the presence of damage-associated molecular patterns (DAMPs), including high-mobility group box 1 (HMGB1), histones, and extracellular vesicles, acts as a bridge between cellular injury and the activation of both immune and thrombotic pathways (2, 3). The integration of DAMP signaling into the immunothrombotic framework reinforces the view of VTE as a systemic immune-mediated event and offers new opportunities for the development of diagnostic biomarkers and immunomodulatory interventions.

Another important aspect is the increasingly recognized role of neutrophils and, specifically, neutrophil extracellular traps (NETs) in VTE pathogenesis. NETs not only provide a physical scaffold for clot formation but also engage directly with the coagulation system. Components such as histones promote platelet activation and thrombin generation, while neutrophil elastase degrades tissue factor pathway inhibitor, further promoting coagulation (4, 5). However, the mechanisms that regulate NET formation, whether through NADPH oxidase-dependent signaling, PAD4-mediated histone citrullination, or mitochondrial pathways, differ across disease contexts and remain incompletely understood (6). The heterogeneity of NETosis mechanisms underscores the importance of targeted approaches in ongoing efforts to develop NET-directed therapies, such as PAD4 inhibitors or recombinant DNase treatments, which are now moving toward early clinical evaluation.

Another recurring and crucial player in the field is the vascular endothelium. In addition to serving as a structural barrier, endothelial cells actively participate in immune surveillance and hemostatic regulation. Inflammatory activation of endothelial cells leads to the upregulation of adhesion molecules such as P-selectin and E-selectin and the release of von Willebrand factor, all of which promote leukocyte and platelet recruitment and facilitate thrombus propagation. Disruption of the endothelial glycocalyx, an early event in thrombus formation, also contributes to these processes by exposing adhesion receptors and altering vascular shear dynamics (7). Recent evidence suggests that preservation of glycocalyx integrity may be a novel therapeutic goal, particularly in patients at high risk for thrombosis.

While innate immune drivers have been extensively studied in the context of VTE, the role of adaptive immunity remains relatively underexplored. Emerging data suggest that Th17 cells induce IL-17-mediated endothelial activation and neutrophil recruitment, which may exacerbate thrombotic risk (8, 9). Conversely, specialized regulatory T cells (Tregs) are involved in venous thrombus resolution (10). Interestingly, B cells may be protective against venous thrombosis as their deficiency promotes DVT through elevated neutrophil counts and fibrinogen levels (11). Greater integration of adaptive immune profiling into thrombosis research may reveal new therapeutic angles, particularly in the context of autoimmune and chronic inflammatory conditions.

A novel and increasingly relevant perspective not explored in depth in this Research Topic, but worth mentioning, is the influence of the gut microbiome on thrombotic risk. Metabolites such as trimethylamine-N-oxide (TMAO), produced by the gut flora, have been shown to increase platelet reactivity and systemic inflammation (12). In conditions such as obesity, diabetes, and aging, gut dysbiosis is associated with chronic low-grade inflammation, a known risk factor for VTE. Investigation of the microbiota–immune–coagulation axis may open new prevention strategies, including dietary modulation or probiotic therapy.

In this Research Topic, Alturky et al. reviewed evidence supporting the connection between metabolic syndrome and post-thrombotic syndrome, identifying key knowledge gaps. Lu et al. reviewed the key role of monocytes and macrophages in the chronic resolution of venous thrombi. Liu et al. identified a significant association between platelet-derived growth factor and venous thromboembolism using a Mendelian randomization-based approach. Cheng et al. highlighted the potential prothrombotic effects of recombinant human granulocyte colony-stimulating factor treatment in patients with cancer. Vincent et al. identified an important role for recombinant mast cell chymase as a negative regulator of endogenous fibrinolysis.

The translational potential of these findings cannot be overstated. As this Research Topic illustrates, targeting inflammatory and immune pathways offers promising avenues for VTE treatment. Statins, for instance, may reduce the burden of venous thrombosis in both experimental and clinical studies, likely by reducing the inflammatory component (13, 14). Additionally, inflammatory biomarkers, such as circulating DNA, IL-6, or soluble P-selectin, may serve as tools for risk stratification, especially in cancer-associated thrombosis where individualized treatment remains a challenge (15). A personalized medicine approach, integrating immunological and coagulation profiles, may ultimately yield better outcomes in both the prevention and management of VTE.

In conclusion, the articles included in this Research Topic advance the understanding of how inflammation and immune responses contribute to venous thromboembolism. By integrating new knowledge in the field, this body of work offers a broader and more nuanced perspective on the pathophysiology of VTE. These contributions not only enhance our current conceptual models but also provide the foundation for novel therapeutic strategies. Continued interdisciplinary collaboration, for example, modeling of venous thrombosis *in silico* (16) or in a vessel-on-a-chip device (17), will be essential to translate these insights from bench to bedside and to fully harness the potential of immunothrombotic research in reducing the burden of VTE.
